# Faculty development and career success in clinical teaching

**DOI:** 10.5116/ijme.693a.e41b

**Published:** 2025-12-22

**Authors:** Anna YuQing Huang, Wan-Yu Yeh, Ezra Jiyang Lin, Jen-Feng Liang, Ying-Ying Yang, Po-Ting Hsu, Chia-Chang Huang, Shiau-Shian Huang, Stephen J.H. Yang, Chen-Huan Chen

**Affiliations:** 1 Computer Science & Information Engineering, National Central University, Taoyuan City, Taiwan; 2 Division of Evidence-based Medicine, Department of Medical Education, Taipei Veterans General Hospital, Taipei, Taiwan; 3 Storage and Content Delivery, Support Engineering, Amazon Web Services, Taipei, Taiwan; 4 Jen-Feng Liang "School of Medicine, National Yang Ming Chiao Tung University, Taipei, Taiwan; 5 Department of Medical Education, Taipei Veterans General Hospital, Taipei, Taiwan

**Keywords:** Faculty development, teaching award, publications, feedback, clinical teachers

## Abstract

**Objectives:**

To explore the effectiveness of overall faculty development (FD) programs in terms of three indicators of successful careers of clinical teachers (CTs): positive feedback (on personality traits or teaching skills) from students, teaching awards, and scholarly publications.

**Methods:**

Data on student feedback, number of teaching awards, number of scholarly publications, and sum of FD participation hours in a teaching hospital with 23 clinical departments and 623 clinical faculty members (2019-2021) were collected and analyzed using Spearman’s rank-order correlation coefficient (r_s_) and independent-samples t-tests (with Welch’s correction where appropriate).

**Results:**

The sum of FD hours was significantly associated with positive feedback from students (r_s_ = 0.15, p = .001) but not with teaching awards or publications. Furthermore, faculty members with more FD hours on research skills received better positive feedback from students regarding personal traits or teaching skills (Cohen’s d = 0.60, 95% CI [0.34, 0.86], p < .001) and more teaching awards (Cohen’s d = 0.34, 95 % CI [0.13, 0.55], p = .010) but did not have a greater number of publications (Cohen’s d = 0.15, 95% CI [-0.07, 0.36], p = .780) than those with lower research FD hours. In addition, the number of teaching awards was significantly associated with positive feedback from students regarding personal traits (r_s_ = 0.92, p < .001) or teaching skills (r_s_ = 0.93, p < .001), and publication quantity (r_s_ = 0.13, p < .001) was markedly correlated with the number of teaching awards.

**Conclusions:**

FD activities may provide positive impacts on CTs in terms of feedback from students and teaching awards but do not directly impact scholarly publications. However, faculty members who received teaching awards and positive feedback from students may have better scholarly publication performance.

## Introduction

Clinical faculty in healthcare multitask as clinicians, researchers, and educators. Apart from delivering clinical services, they aim to publish academic research and offer clinical education to undergraduate or postgraduate trainees. The dual commitment to academic and teaching excellence presents a formidable challenge as clinical faculty navigate the intricate balance between research and teaching demands.

To help clinical faculty face such challenges, faculty development (FD) units have increased in the past two decades. The main topics of FD cover all competencies needed by clinical faculty, such as teaching and evaluation skills, mentoring, leadership, and research related issues.^1^^,^^2^ Possessing reserved time for participating in FD activities relevant to their work makes faculty members feel valued and encouraged.^3^ However, the FD activities designed to help clinicians may add work for busy faculty members.

Additionally, the FD programs are resource-intensive for the institution. The effectiveness of FD programs should be well-evaluated and confirmed.^4^

In many teaching hospitals, a variety of teaching skill courses have been provided and reported for developing literacies as a competent clinical teacher. Some studies have demonstrated that faculty members established personal charisma, gained positive feedback from students, or had higher post-test scores related to teaching skills after receiving FD training.^1^^,^^5^ Considering the role of researchers, several reports on FD activities designed for enhancing research ability, such as input/assets, teamwork building, project planning, systemic processes, and scholarly maturation, indicated promising results in increasing the publishing productivity of clinical faculty, which enhances their sense of achievement in academic work.^6^ Nevertheless, the effectiveness evaluation of many FD activities usually focused on a lower level of Kirkpatrick’s hierarchy (e.g., course satisfaction as well as knowledge of education/research related skills) and lacked long-term follow-up.^7^^-^^10^ To evaluate the higher level effectiveness indicators, such as behavior change or return on investment, FD activity is crucial for faculty development plans. In addition, most studies on FD focused on single or several programs instead of a large-scale panorama of the impact of FD on the institution.

Several indicators may be applied to evaluate the faculty. Among them, student evaluation on teaching (SETs) and teaching awards were considered the most important^11^^,^^12^ because feedback from students provided direct information about teaching quality, whereas winning teaching awards may be considered an indicator of successful faculty career development. When considering academic performance, a number of scholarly publications are typically considered in many instances, such as faculty evaluations, grant applications, and promotions. Positive feedback from students, teaching awards, and more scholarly publications may be considered features of successful careers among clinical faculty and could serve as indicators of higher Kirkpatrick-level of FD training effectiveness (behavior and result change).

Because higher-level objective effectiveness evaluations about large-scale FD implementation is limited, this study aimed to evaluate the overall effectiveness of FD program by analyzing the relationship among FD participation and the above three indicators of successful clinical career. The research questions include: 1. What is the correlation among the participation of faculty development, teaching award, students’ feedback, and scholarly publications? 2. Is there any difference in students' feedback, teaching awards, and scholar publications according to the level of FD participation? 3. Is there any difference in scholarly publications, FD program hour, feedback of students, and teaching awards between different specialties? By analyzing the established hospital faculty database, this study will provide objective evidence of the higher Kirkpatrick-level effectiveness of overall FD program effectiveness and references for FD program design in the future.

## Methods

### Study design and participants

The present study is a secondary analysis using de-identified data, so IRB approval has been waived according to the regulation of our hospital. In this study, the data of total clinical faculty (n=623) from 23 clinical departments, all of them were physicians or dentists, was collected from one of the major teaching hospitals in Taiwan.

### Data collection method

The database was extracted from the electronic database of the study hospital, including participation in FD activities, academic publications, and teaching awards winning status on all clinical faculty from 2019-2021. These data are routinely collected and reviewed for teaching improvement periodically. The feedback for clinical teachers (CTs) from students was extracted from the teaching-assessing system (TAS), which was launched in 2012 in the study hospital.

### Instrument and Study Setting

Since student evaluations of teaching (SETs) are an important source of teacher evaluation, we developed this feedback tool initially in 2012. The two categories “personal trait” (teacher’s attitude in teaching) and “teaching skills” are designed intentionally in our tool at time of questionnaire development rather than subcategorized during this study. The details of the questions are provided in the Appendix. The validity (CVI) of this questionnaire is 0.94. The contents of these questions have been reviewed again in 2017 (due to MD curriculum change) and was not modified at that time. The teaching awards include the “annual clinical teaching excellence award” and “annual student-voted best teaching award” and were viewed as the indicative and representative honor of clinical faculty contributing to the clinical medical education field. There are approximately 15-20 winners every year for each award. The teaching awards are largely decided by the voting of the trainees, and the CTs could win the award consecutively. We extracted the number for winning the two awards among all CTs during the study period for analysis.

Four categories of FD programs—teaching skills (counseling skills, course design, evaluation skills, instruction skills, teaching material development), research/paper writing skills, medical ethics and law, and leadership skills—were provided in this teaching hospital, and all clinical faculty were asked to participate at least four hours of teaching skill training annually. We examined the CTs’ time investment in FD activities according to teaching skills hours, research skills hours, and the total hours of FD participation. For academic publications, we used the number of clinical faculty’s annual publications as references.

The fields of clinical faculty’s publications are determined by their personal expertise and are not limited to publications in education-related fields but mostly focus on papers in the professional field of clinical medicine. Since the faculty development courses are designed to enhance all competencies of clinical staff as a faculty, the research FD courses are focused on enhancing the scholar ability, not only educational but basic or clinical, among all clinical teachers. In this study, we want to explore if the faculty who participate in more teaching skill training have better academic output. We hypothesize that teaching helps inspire research ideas and find research team members.

### Variables

After data cleaning by removing mismatched or incomplete data and excluding records with student-feedback responses below the 25th quartile of total student counts, our study employed a two-phase process for data handling as follows:

#### Phase 1: Defining Variables

The study examined FD participation across three dimensions: (1) Academic Ability: Defined as the total hours of participation in research skill programs, representing the faculty’s engagement in academic development. (2) Teaching Capability: Defined as the total hours spent in teaching and mentoring skill development activities. (3) Total FD Participation: The sum of both academic ability and teaching capability. Additionally, the study included data on teaching awards. Each faculty member's award status was recorded: a value of 1 was assigned for winning an award and 0 otherwise. Teaching award data was further combined with scores from the ninth item on the student feedback questionnaire to enhance the assessment of faculty teaching effectiveness. Faculty members who won more than two teaching awards were excluded from this analysis due to the small sample size (n=6), which could introduce statistical bias.

The dependent variables were as follows: (1) Student feedback: The total annual score of student evaluations for each faculty member from 2019-2021. This was subdivided into two categories: feedback on personal traits and feedback on teaching skills. (2) Scholarly publications: The annual total of academic publications by each clinical faculty member from 2019-2021.

#### Phase 2: Grouping Variables

The defined variables were grouped for analysis:

By participation level: Numeric variables, including academic ability (annual hours), teaching capability (annual hours), total FD participation (sum of both), teaching awards, student feedback scores (divided into two aspects─personality traits/teaching skills), and academic publications, were categorized into high (top 25%, above the third quartile) and low (bottom 25%, below the first quartile) groups based on their distribution.

By award status: Faculty members were divided into two groups: award recipients and non-recipients. Any faculty member who received at least one teaching award was included in the recipient group; those without awards were classified as non-recipients.

By department: Faculty were also categorized based on their department affiliation, grouped into either internal medicine or surgery, with internal medicine marked as the reference group.

### Data Analysis

Statistical analyses were performed using SPSS v20. Spearman’s rank-order correlation coefficient (rs) was used to examine associations among FD participation, teaching awards, student feedback, and scholarly publications (n = 623). Group comparisons were conducted using independent-samples t-tests. Levene’s test was used to assess equality of variances; when variances were unequal, t-values and degrees of freedom from Welch’s correction were reported. For all t-tests, means and standard deviations (M ± SD), the t statistic with degrees of freedom, exact p-values, and Cohen’s d were reported to indicate effect size. All p-values are reported to three decimal places (except p < .001). Statistical significance was set at p < .05.

## Results

The correlations among participation of FD, feedback from students, and scholarly publications are shown in Table 1. Spearman’s correlation coefficient (rs) indicated that participation in research skill FD programs was significantly positively correlated with student feedback regarding personal traits (rs = 0.20, p < .001) and teaching skills (rs = 0.19, p < .001). Total FD participation hours were also significantly correlated with both aspects of student feedback (rs = 0.15, p = .002 and rs = 0.15, p = .004, respectively). In addition, teaching awards among clinical faculty were significantly positively correlated with academic publications (rs = 0.13, p < .001) and student feedback on both personal traits (rs = 0.92, p < .001) and teaching skills (rs = 0.93, p < .001).

The results of the differences between students' feedback, teaching awards, and academic publications, according to the participation level of different faculty development topics, are shown in Table 2. We divided all faculty into high and low groups according to the research skill FD hours and denoted them by the symbols GH (Research) and GL (Research).

Research-skill FD hours. Faculty in the higher-hours group for research skills (GH [Research]) had higher student-feedback scores than those in the lower-hours group (GL [Research]). For personal traits feedback, GH (n = 109) had M = 51.01 (SD = 12.26) versus GL (n = 124), M = 41.98 (SD = 17.01), t(222.74) = 4.68, p < .001, Cohen’s d = 0.60, 95% CI [0.34, 0.86]. For teaching skills feedback, GH (n = 109) had M = 50.68 (SD = 12.13) versus GL (n = 124), M = 41.97 (SD = 16.96), t_(__222.14)_ = 4.55, p < .001, Cohen’s d = 0.58, 95% CI [0.32, 0.85]. GH (Research) also had more teaching awards than GL (Research): GH (n = 157), M = 9.19 (SD = 6.69) versus GL (n = 195), M = 6.95 (SD = 6.32), t_(__350) _= 3.21, p = .001, Cohen’s d = 0.34, 95% CI [0.13, 0.55]. However, the number of academic publications did not differ significantly between GH (Research) and GL (Research): GH (n = 157), M = 13.30 (SD = 22.51) versus GL (n = 195), M = 10.58 (SD = 16.22), t_(241)_ = 0.78, p = .436, Cohen’s d = 0.15, 95% CI [−0.07, 0.36].

**Table 1:** The correlation matrix among variables of faculty development hours, teaching award winning, students feedback and scholarly publication amount

Variables	(1)	(2)	(3)	(4)	(5)	(6)	(7)
(1) Total hours of Research skills training	1						
(2) Total hours of Teaching skills training	.35	1					
(3) Sum of faculty development hours	.72	.87	1				
(4) Teaching award winning (times)	.11	.06	.10	1			
(5) Feedback from students: Personal traits	.20***	.06	.15^**^	.92***	1		
(6) Feedback from students: Teaching skills	.19***	.07	.15**	.93***	.98	1	
(7) Academic publication	.06	-.02	.01	.13***	.21	.21	1

Spearman’s rank-order correlation coefficient (*r_s_*), *n* = 623. **significant at *p* < .01, ***significant at *p* < .001

**Table 2:** The difference of FD hours, receiving teaching award, students’ feedback and scholarly publication amount according to the research skill FD hours, teaching skill FD hours and total FD hours

FD type	Variables		Group (n)	Mean ± SD	*t*_(df)_	*p*	Cohen's *d*[95% CI]
**Research skills**	Students’ feedback	Personality trait	G_H_ (109)	51.01 ± 12.26	4.68 (222.74)	<.001	**0.60 [0.34, 0.86]**
			G_L_ (124)	41.98 ± 17.01			
		Teaching skill	G_H_ (109)	50.68 ± 12.13	4.55 (222.14)	<.001	**0.58 [0.32, 0.85]**
			G_L_ (124)	41.97 ± 16.96			
	Scholarly publication		G_H_ (157)	13.30 ± 22.51	0.78 (241)	.436	**0.15 [-0.07, 0.36]**
			G_L_ (195)	10.58 ± 16.22			
	Teaching award winning		G_H_ (157)	9.19 ± 6.69	3.21 (350)	.001	**0.34 [0.13, 0.55]**
			G_L_ (195)	6.95 ± 6.32			
**Teaching skills**	Students’ feedback	Personality trait	G_H_ (100)	47.22± 15.22	0.95 (203)	.344	**0.13 [-0.14, 0.41]**
			G_L_ (105)	45.13± 16.19			
		Teaching skill	G_H_ (100)	46.99 ± 15.10	0.99 (203)	.324	**0.14 [-0.13, 0.42]**
			G_L_ (105)	44.80 ± 16.04			
	Scholarly publication		G_H_ (157)	12.63 ± 19.42	0.20 (236)	.842	**0.02 [-0.20, 0.23]**
			G_L_ (181)	12.32 ± 17.81			
	Teaching award winning		G_H_ (157)	7.80 ± 6.67	1.41 (336)	.158	**0.15 [-0.06, 0.36]**
			G_L_ (181)	6.77 ± 6.58			
**Total FD hours**	Students’ feedback	Personality trait	G_H_ (106)	49.09 ± 13.93	3.30 (197.89)	.001	**0.48 [0.20, 0.76]**
			G_L_ (91)	41.61 ± 17.40			
		Teaching skill	G_H_ (106)	48.81 ± 13.83	3.31 (172.26)	.001	**0.48 [0.19, 0.76]**
			G_L_ (91)	41.38 ± 17.19			
	Scholarly publication		G_H_ (160)	14.72 ± 24.77	1.23 (225)	.219	**0.16 [-0.06, 0.38]**
			G_L_ (157)	11.38 ± 16.14			
	Teaching award winning		G_H_ (160)	8.44 ± 6.72	2.95 (315)	.003	**0.32 [0.10, 0.54]**
			G_L_ (157)	6.27 ± 6.35			

FD: Faculty Development, GH: Higher-hours group, GL: Lower-hours group, SD: standard deviation, CI: confidence interval.

*t*_(df)_ = independent-samples t statistic and degrees of freedom (Welch’sdf reported where variances were unequal). Cohen’s *d* reported with 95% confidence intervals.

Teaching-skill FD hours. When faculty were grouped by teaching-skill FD hours (GH [Teaching] vs GL [Teaching]), no significant differences were observed in student feedback, teaching awards, or publication counts. Specifically, for personal traits feedback, GH (n = 100), M = 47.22 (SD = 15.22) versus GL (n = 105), M = 45.13 (SD = 16.19), t(203) = 0.95, p = .344, Cohen’s d = 0.13, 95% CI [−0.14, 0.41]; for teaching skills feedback, GH (n = 100), M = 46.99 (SD = 15.10) versus GL (n = 105), M = 44.80 (SD = 16.04), t_(203)_ = 0.99, p = .324, Cohen’s d = 0.14, 95% CI [−0.13, 0.42]. Scholarly publication counts did not differ (GH [n = 157], M = 12.63, SD = 19.42 vs. GL [n = 181], M = 12.32, SD = 17.81), t_(236)_ = 0.20, p = .842, Cohen’s d = 0.02, 95% CI [−0.20, 0.23], nor did teaching award counts (GH, M = 7.80, SD = 6.67 vs. GL, M = 6.77, SD = 6.58), t_(336)_ = 1.41, p = .158, Cohen’s d = 0.15, 95% CI [−0.06, 0.36].

Total FD hours. Grouping by total FD participation (GH [Total] vs GL [Total]) revealed significant differences in student-feedback scores and teaching awards but not in publication counts. For personal traits feedback, GH (n = 106), M = 49.09 (SD = 13.93) versus GL (n = 91), M = 41.61 (SD = 17.40), t_(197.89)_ = 3.30, p = .001, Cohen’s d = 0.48, 95% CI [0.20, 0.76]; for teaching skills feedback, GH (n = 106) M = 48.81 (SD = 13.83) versus GL (n = 91) M = 41.38 (SD = 17.19), t_(172.26) _= 3.31, p = .001, Cohen’s d = 0.48, 95% CI [0.19, 0.76]. The number of teaching awards was greater in GH (Total) than in GL (Total): GH (n = 160), M = 8.44 (SD = 6.72) versus GL (n = 157), M = 6.27 (SD = 6.35), t_(__315)_ = 2.95, p = .003, Cohen’s d = 0.32, 95% CI [0.10, 0.54]. Scholarly publication counts did not differ significantly between GH (Total) and GL (Total) (GH, M = 14.72, SD = 24.77 vs. GL, M = 11.38, SD = 16.14), t_(__225)_ = 1.23, p = .219, Cohen’s d = 0.16, 95% CI [−0.06, 0.38].

**Figure 1:**
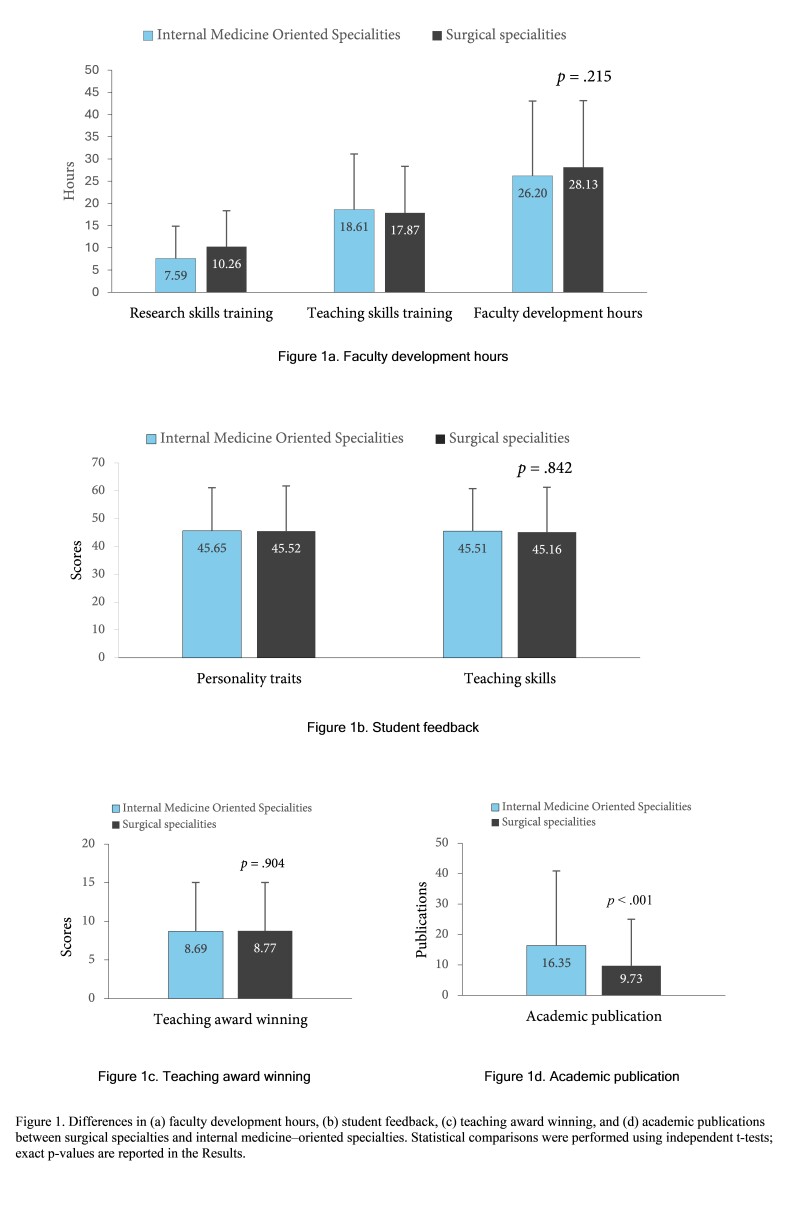
Differences in (a) faculty development hours, (b) student feedback, (c) teaching award winning, and (d) academic publications between surgical specialties and internal medicine–oriented specialties. Statistical comparisons were performed using independent t-tests; exact p-values are reported in the Results.

Figure 1 summarizes comparisons between surgical and internal-medicine–oriented specialties. As depicted in Figure 1a, the surgical faculty reported more research skill FD hours (M = 10.26, SD = 8.09) than internal medicine faculty (M = 7.59, SD = 7.31), Welch’s t_(__381.48) _= 3.69, p < .001, Cohen’s d = 0.35, 95% CI [0.17, 0.53]. Student feedback scores did not differ significantly between the two specialty groups for either dimension. Personal traits feedback scores were similar between internal medicine (M = 45.65, SD = 15.60) and surgical faculty (M = 45.52, SD = 16.24), t_(367)_ = 0.18, p = .945, Cohen’s d = 0.02, 95% CI [−0.19, 0.23]; teaching skills feedback was likewise comparable (M = 45.51, SD = 15.42 vs. M = 45.16, SD = 16.12), t_(367)_ = 0.30, p = .842, Cohen’s d = 0.03, 95% CI [−0.18, 0.24] (Figure 1b).

The number of teaching awards was also similar between internal (M = 8.69, SD = 6.36) and surgical medicine faculty (M = 8.77, SD = 6.26), t_(__501)_ = 0.186, p = .904, Cohen’s d = 0.02, 95% CI [−0.17, 0.19] (Figure 1c). In contrast, the internal medicine faculty had a significantly greater number of academic publications than the surgical faculty (M = 16.35, SD = 24.25 vs. M = 9.73, SD = 16.56), Welch’s t_(__360.99)_ = 3.52, p < .001, Cohen’s d = 0.35, 95% CI [0.13, 0.56] (Figure 1d).

## Discussion

This study explored the association among FD hours, feedback from students regarding CTs, teaching awards, and scholarly publication performance among clinical faculty. The results highlighted that FD hours had a positive association with feedback scores from students. The group that participated in most FD activities had better student feedback and won more teaching awards than those that participated in fewer FD activities. While considering scholarly publication, interestingly, it only correlated to teaching award winning in our study.

Faculty development is expected to increase the teaching literacy of clinical faculty. However, there is a merely modest effect on feedback from medical students in this study. This is probably because a basic amount of faculty development training is needed to improve the teaching skill. In other words, there is a threshold effect of FD training. The argument is supported by the group that participated in the most FD activities and performed better than those who participated in fewer FD activities (below the 25^th^ percentile). Our result implies that an essential FD requirement may be important. Another potential reason is the delay effect of learning. Faculty need time to digest and internalize the learned skills before demonstrating an increase in teaching skills; therefore, our study fails to show the improvement due to this time gap.

In our study, the research skill FD activities played a stronger role than teaching skills in influencing student feedback and teaching awards. We believe that some research skill topics, such as clinical research introduction and academic writing, are attractive to clinical trainees. CTs familiar with these topics can better satisfy the learning needs of the trainees. Moreover, the form and topics of FD programs are more diverse than research skills. Some are basic and addressed in a big hall, whereas some are more advanced and held in small-group workshops. Our result does not overthrow the importance of teaching skill training. Instead, it establishes that faculty development program planners should customize the teaching skill according to the needs of different learners and evaluate the effect of the individual program rather than overall programs.

In our study, teaching awards were associated with the feedback of students. To win a teaching award required more experience and integration of different teaching skills,13 so the short-term effect of FD on teaching awards was insignificant in this cross-sectional study. However, we believe that FD activities are helpful in teaching awards because the FD activities are associated with the positive feedback of students. More research should be conducted to validate our argument.

As an important indicator while evaluating the effect of FD activities, there was no difference between the higher or lower FD hours group regarding the number of scholarly publications. Similar to the above discussion, the scholarly publication needs time to process.^14^ It could serve as a long-term indicator of FD activities. Some alternative indicators, such as the number of research proposals, oral presentations, and winning grants, should be considered when evaluating the effect of FD in terms of academic career.^15^ Interestingly, faculty members with more scholarly publications had higher feedback scores from students and more teaching awards. A higher number of scholarly publications is related to a higher academic reputation. We speculated that the students might give higher scores to faculty with more publications because these teachers often earn respect among colleagues and are role models for students, i.e., the halo effect.^16^ Similar cases may exist in decisions regarding teaching awards. Faculty evaluators should observe the impact of academic reputation on teaching evaluation.

It is important for FD program planners to understand the target learners’ needs. Different specialties have different working schedules, priorities, teaching and academic work, culture, and resources. Not surprisingly, internal medicine teachers had more academic publications.^17^ However, surgical CTs participated in more research skill FD courses, probably because the surgical doctors were busier with clinical work and had less time to learn from colleagues. The results are important for customized FD program planning.

This study’s strength lay in utilizing three-year primary objective data for analysis. Application of a quantitative statistical method brought more convincing evidence. Additionally, indicators of higher Kirkpatrick levels were evaluated, such as the behavior (feedback from the students) and results (award winning and publication).

However, this study had a few limitations. First, the cross-sectional design of this study could hardly present the causal relationship of each variable, especially those long-term outcomes. Second, this was a single-centered based analysis and may be less representative. The FD programs, rules, and institutional culture should be variable in different contexts. Besides, we cannot rule out that teachers who actively participate in FD may themselves be more enthusiastic about teaching, and therefore may have better feedback from students. In addition, although students' feedback on teachers' behavior provides one important measure of teachers' learning effectiveness in FD programs that cannot be evaluated by others, but student evaluations of teaching do not represent a completely fair evaluation of CTs' teaching quality.^18^^,^^19^ While the aforementioned concerns affect how the study's conclusions should be interpreted, owing to considerable faculty numbers and long period of our database, this study is noteworthy to be reported as an example of objective overall institutional FD effectiveness evaluation.

## Conclusions

Faculty development activities positively affect CTs regarding feedback from students and teaching awards winning. However, this did not apply to scholarly publications during the three-year observational period. Nevertheless, faculty members who received teaching awards and positive feedback from students may have better scholarly performance. In addition, the needs of FD activities may be different according to specialty. Faculty development activities do show their value for CTs and are worthy of institutional support. Further research is needed to validate these findings and explore the impact of FD programs on students and the institutions.

### Acknowledgements

This research was supported by the grants of the Taipei Veterans General Hospital, Taipei, Taiwan (Grant No.: VTA115-V1-6-1& V115C-023, V115EA-004), National Yang Ming Chiao Tung University (114Q159502) and National  Science and Technology Council, Taiwan (Grant No.: NSTC 112-2314-B-A049-043-MY3; NSTC 114-2410-H-A49 -029 -MY2). The funders had no role in study design, data collection and analysis, decision to publish, or preparation of the manuscript.

### Conflict of Interest

The authors declare that there is no conflict of interest.

## Supplementary material

Supplementary file 1 Appendix. The Questions List of Student Questionnaires
