# Supplementary material for: Faculty development and career success in clinical teaching
Source: Int J Med Educ. 2025 Dec 22;16:203–11. doi: 10.5116/ijme.693a.e41b (PMC12768577; doi:10.5116/ijme.693a.e41b)
Supplement: Supplementary file 1 — Appendix. The Questions List of Student Questionnaires [file ijme-16-203-S1.pdf]

Appendix

The Questions List of Student Questionnaires

Categories	Description of Questions
**Personal Traits (PT)**	*(PT) Question 2: My clinical teacher is enthusiastic about teaching and could trigger my interest in this department.*
	(PT)Question 3: My clinical teacher is concerned about my learning condition and would give some suggestions properly.
	(PT) Question 5: My clinical teacher act as my backup strongly, which makes me learn from clinical practice smoothly.
	(PT) Question 8: My clinical teacher is a great role model or a role that I would like to become in the future.
**Teaching Skills (TS)**	*(TS) Question 1: My clinical teacher can easily manifest our target and anticipation according to our training status clearly.*
	(TS) Question 4: In addition to professional knowledge, clinical teacher could give me some advice in medical law, ethics, communication, and holistic care aspect.
	(TS) Question 6: My clinical teacher gives me feedback according to my performance.
	(TS) Question 7: My clinical teacher teaches and guides me on medical record writing.
**Overall**	*(Overall) Question 9: Overall, my clinical teacher could help my clinical learning effectively.*
